# Effects of Seawater Acclimation on Serum Biochemistry, Hormones, Splenic Immunity, Hepatic Lipid Metabolism, and Intestinal Microbiota in the F_2_ Generation of Chinese Sturgeon (*Acipenser sinensis*)

**DOI:** 10.3390/ani16142204

**Published:** 2026-07-15

**Authors:** Xing Chen, Wei Xiong, Min Zhao, Jinping Wu, Xijun Hu, Runze Jin, Pei Zhang, Hao Du, Yuan Liu, Hanwen Yuan

**Affiliations:** 1 College of Animal Science and Technology, Yangtze University, Jingzhou 434023, China; 2 Key Laboratory of Freshwater Biodiversity Conservation, Ministry of Agriculture and Rural Affairs of China, Yangtze River Fisheries Research Institute, Chinese Academy of Fishery Sciences, Wuhan 430223, China; 3 Shandong New Hope Liuhe Group Co., Ltd., Qingdao 266061, China; 4 Aquatic Industry Development Center, Huangmei Bureau of Agriculture and Rural Affairs, Huanggang 435500, China; 5 Hubei Fisheries Industrial Technology Research Institute, Jingzhou 434000, China

**Keywords:** Chinese sturgeon (*Acipenser sinensis*), salinity, serum biochemistry, lipid metabolism, splenic immunity, intestinal microbiota

## Abstract

This study aimed to investigate the effects of salinity changes on serum biochemistry, hormonal profiles, splenic immune function, hepatic lipid metabolism and intestinal microbiota of the F_2_ generation of Chinese sturgeon (*Acipenser sinensis*) under freshwater and seawater acclimation (15–23.2 psu) conditions. A 30-day indoor culture experiment showed that seawater acclimation only induced a declining trend in juvenile growth without statistical significance compared to the freshwater group (*p* > 0.05). The levels of triglycerides (TG), total cholesterol (TC), low-density lipoprotein cholesterol (LDL-C), alkaline phosphatase (ALP) and albumin II (ALBII) in the freshwater group were markedly lower than those in the seawater group (*p* < 0.05), while alanine aminotransferase (ALT), aspartate aminotransferase (AST), high-density lipoprotein cholesterol (HDL-C) and total protein (TPII) showed no significant differences (*p* > 0.05). The serum triiodothyronine (T3) concentration was significantly higher, and chloride ion (Cl^−^) was lower in freshwater individuals (*p* < 0.05). Cortisol, testosterone, K^+^, Na^+^, Ca^2+^, thyroid-stimulating hormone and T4 remained unchanged between the two salinity environments (*p* > 0.05). In gill tissues, lipase (LPS) and fatty acid synthase (FAS) activities were lower, whereas lipoprotein lipase (LPL) activity was higher in the freshwater group (*p* < 0.05), and no obvious difference was observed in hepatic metabolic enzymes. The splenic complement C4 content was significantly decreased in freshwater sturgeon (*p* < 0.05), while splenic C3, IgM and serum immune indices showed no statistical differences. All alpha-diversity indices of intestinal microbiota were significantly higher in the seawater group (*p* < 0.05). At the phylum level, the intestinal flora in the seawater group was dominated by *Proteobacteria* (69.6%) and *Fusobacteriota* (14.9%), whereas *Fusobacteriota* accounted for 81.5% in the freshwater group. At the genus level, seawater samples exhibited richer microbial composition including *Sphingomonas*, *Cupriavidus* and *Cetobacterium*, while the freshwater microbiota was overwhelmingly dominated by *Cetobacterium* (81.5%). In conclusion, salinity significantly regulates serum biochemistry, hormone secretion, lipid metabolism, splenic immunity and intestinal microbiota of the F_2_ generation of Chinese sturgeon. The freshwater environment benefits lipid metabolism, thyroid hormone levels, and growth performance, with a simplified intestinal microbial structure. Seawater acclimation maintains higher gut microbial diversity and immune homeostasis, but induces osmotic stress, restricts thyroid hormone secretion and redistributes energy supply, thereby inhibiting fish growth. This study provides a theoretical basis for salinity domestication and scientific culture management of Chinese sturgeon.

## Simple Summary

The Chinese sturgeon (*Acipenser sinensis*) is a fish species facing the risk of extinction. When bred in freshwater ponds, most farmed individuals cannot reach sexual maturity or reproduce normally. In the wild, these fish migrate to the ocean, so moving them into seawater is thought to improve their growth. Still, we know little about how saltwater changes their physical condition. In this research, we raised young Chinese sturgeon in freshwater and seawater separately for 30 days and tested a series of physical indicators including blood status, growth-related hormones, immune performance, fat processing in their bodies, and gut bacteria (tiny microbes living in their intestines). We found sturgeon living in freshwater gained a little more weight and produced more growth-promoting hormones. Those kept in seawater had more diverse gut microbes and showed immune adjustments in their spleen (an important immune organ of fish), yet their growth slowed down. Our results clarify the different influences of freshwater and seawater on young Chinese sturgeon, and provide practical guidance for artificial breeding to protect this rare endangered fish and restore its wild populations.

## 1. Introduction

The Chinese sturgeon (*Acipenser sinensis*) is a large anadromous fish with an extremely complex life history. Due to threats including overfishing, water pollution, hydraulic engineering, and shipping activities, the wild population of the Chinese sturgeon has become critically endangered and was classified as Critically Endangered (CR) by the International Union for Conservation of Nature (IUCN) in 2010 [1,2]. Captive freshwater-reared populations commonly exhibit prolonged gonadal arrest at stage II, poor oocyte quality, and smaller body size, which severely constrains large-scale artificial propagation and stock restoration [3]. Long-term freshwater rearing, characterized by low ambient osmotic pressure, readily disrupts osmoregulation and ion homeostasis in the Chinese sturgeon [4]. Although juveniles can adapt to freshwater environments by modulating the width and spacing of gill lamellae, prolonged freshwater acclimation reduces metabolic capacity and diminishes tolerance to high-salinity seawater environments [5]. Therefore, exploring the physiological responses of the Chinese sturgeon under salinity changes is of great significance for artificial domestication, resource conservation and release enhancement.

Salinity is one of the most critical environmental factors affecting the growth, metabolism, immunity, and gut microbiota of euryhaline fishes [6]. Abrupt salinity fluctuations require substantial energy expenditure for ion transport and the reconstruction of osmoregulatory systems in gill and renal tissues [7]. Salinity stress alters energy allocation strategies in fish, suppresses somatic growth, disrupts lipid metabolism, and reshapes gut microbial community structure [8]. Previous studies have demonstrated that ambient salinity can significantly modify biochemical parameters, endocrine hormone secretion, and lipometabolic enzyme activities in aquatic animals [9]. Low-salinity environments tend to maintain relatively stable metabolic rates and lower lipid accumulation, whereas high-salinity seawater induces physiological compensation and metabolic adaptation [10]. Furthermore, salinity influences intestinal osmotic pressure, mucosal secretion, and host immune status, thereby further selecting for and reshaping gut microbial composition [11]. As a typical euryhaline species, the Chinese sturgeon can survive across a broad salinity range; however, salinity transitions inevitably incur additional energy costs and limit growth performance [12]. Appropriate salinity is essential for healthy growth and reproduction, whereas inappropriate salinity stress can lead to growth retardation, immunosuppression, and metabolic disorders [8]. Notably, in many euryhaline fishes, the relationship between salinity and growth follows a “U-shaped” or “inverted V-shaped” pattern: intermediate salinity conditions generally favor growth, whereas extremely high or low salinities suppress growth [13]. Studies on spotted scat (*Scatophagus argus*) juveniles have revealed an energy trade-off between salinity and growth: although feed intake increases with rising salinity, growth performance does not increase proportionally, with optimal growth achieved at moderate salinity (15 psu) [14]. Similarly, research on olive flounder (*Paralichthys olivaceus)* juveniles found that salinities ranging from 12 to 40 psu had no significant effect on wet-weight growth in juveniles averaging 2.6 g, whereas salinities of 5 psu and 47 psu markedly impeded growth [15]. A 180-day chronic salinity stress study (9 psu) on largemouth bass (*Micropterus salmoides*) demonstrated that the seawater group exhibited significantly lower growth performance than the freshwater control; however, the seawater group still showed a certain compensatory growth trend in the later stage (180 d) [16]. These findings suggest that the effects of salinity stress on fish growth are both species-specific and closely related to stress intensity and acclimation duration.

This study systematically analyzed the differences in serum biochemistry, hormones, splenic immunity, hepatic-gill lipid metabolism and intestinal microbiota between freshwater and seawater-acclimated F_2_ generation of Chinese sturgeon, and emphatically explored the intrinsic mechanism of growth decline under seawater stress, aiming to provide scientific reference for salinity domestication and refined culture of Chinese sturgeon.

## 2. Materials and Methods

### 2.1. Diet Formulation

In this study, imported Peruvian fishmeal, chicken meal, and squid viscera meal were used as the primary animal protein sources; dehulled soybean meal served as the main plant protein source, wheat flour acted as the main carbohydrate source, and fish oil and purified ARA oil were the main lipid sources. The formulation and nutritional composition of experimental diets are shown in Table 1.

All diet ingredients were ground to ensure uniform particle size. The ground ingredients were then sieved through a 60-mesh standard sieve to remove coarse particles, ensuring uniformity in subsequent mixing and pelleting processes. According to the diet formulation, each ingredient component was accurately weighed using an electronic balance. For trace additive components (such as multivitamins), a stepwise pre-mixing method was employed (i.e., initially mixing with a small amount of carrier and then gradually increasing the mixing ratio) to ensure their uniform distribution in the diet. Fish oil and purified ARA oil were slowly added to the mixer and thoroughly stirred with the other dry ingredients to ensure uniform coating of the oil on the particle surfaces. Subsequently, 20% water was added, and stirring continued until a homogeneous wet mixture was formed. The mixed diet was pelleted into uniformly shaped cylindrical particles using a pellet mill, ensuring an appropriate level of particle compactness to minimize powdering. The wet pellets were spread out evenly in a well-ventilated indoor area, placed in a cool and dry environment, and an electric fan was turned on to accelerate diet drying. The dried diet pellets were then sealed in packages and transferred to a low-temperature freezer at −20 °C for storage.

### 2.2. Experimental Fish and Husbandry Management

The experimental subjects in this study were the F_2_ generation of Chinese sturgeons artificially bred at the Taihu Experimental Station of the Yangtze River Fisheries Research Institute, Chinese Academy of Fishery Sciences, with an initial average body weight of 2.59 ± 0.62 kg. Prior to the experiment, all individuals underwent rigorous health screening to ensure they were free from diseases and abnormal behaviors. The experiment was set up with two groups: the MF group (freshwater group) and the MS group (seawater acclimation group). For the MS group, a gradient acclimation method was employed for salinity adaptation, with the water salinity gradually increased by 1 psu per day until reaching the target salinity of 20 psu. The selection of this salinity range was based on three considerations: (1) literature evidence that Chinese sturgeon juveniles possess strong osmoregulatory capacity and high survival rates within 15–23.2 psu [5]; (2) ecological relevance to the natural salinity experienced by Chinese sturgeon during the fattening period in the Yangtze estuary and coastal waters; and (3) the target salinity of 20 psu near the isosmotic point (approximately 9.52 psu) with measured daily fluctuations of 15–23.2 psu falling within normal water quality variation. The entire acclimation process lasted approximately 20 days to ensure the physiological adaptability of the experimental fish. After reaching the target salinity, the fish were further reared at 20 psu for 30 days as the formal experimental period.

The experiment was conducted in indoor oval-shaped cement rearing tanks (3 m in diameter and 0.75 m in water depth), with one tank per treatment group and 14 fish per tank. Due to the large size of Chinese sturgeon (average 2.6 kg) and limitations of experimental conditions, animal ethics, and sample availability, this design is consistent with current conventional protocols for physiological studies on large endangered fish species, and similar designs have been employed in previous studies [5,17]. Before the formal experiment, all F_2_ generation Chinese sturgeons were temporarily reared in this environment for 14 days to acclimate to the experimental conditions. The diet was administered using the apparent satiation method, with a homemade experimental diet fed twice daily, in the morning and evening, at a daily dieting rate of approximately 1% of the fish’s body weight, dynamically adjusted according to the actual dieting situation to avoid residual diet polluting the water quality. During the rearing period, the water temperature ranged from 21.2 to 24.8 °C, with dissolved oxygen levels ≥ 5.56 mg/L. Sewage was discharged once daily, with approximately two-thirds of the tank water being drained to ensure water cleanliness and reduce the accumulation of ammonia nitrogen and nitrite.

### 2.3. Sample Collection and Measurement

After the rearing experiment, all experimental fish were fasted for 24 h to ensure the emptying of the digestive tract and reduce interference from food residues in the subsequent determination of physiological indicators. Subsequently, all 14 fish from each tank were randomly captured by random netting for body weight and length measurements. Then, 8 fish were randomly selected from each group using a random number table for dissection and further physiological and biochemical indicator measurements. The experimental fish were anesthetized with MS-222. Once the fish were fully anesthetized (with slow and regular gill cover movements), the body weight and length of each fish were measured. After wiping the fish body surface dry with sterile gauze, blood was collected using the caudal vein sampling method with a 5 mL disposable syringe. The blood samples were immediately placed in a 4 °C refrigerator for 12 h for natural clarification. They were then centrifuged at 3000 g and 4 °C for 15 min in a low-temperature centrifuge, and the supernatant was aliquoted into 1.5 mL EP tubes. The serum samples were temporarily stored in a −20 °C refrigerator for subsequent antioxidant indicator detection. The gill covers were opened to collect gill filaments, and the fish were dissected to expose the internal organs in sequence. The spleen and liver tissues were completely stripped, rinsed with pre-cooled physiological saline, and tissue blocks of the spleen and liver were taken. The posterior intestinal segment (approximately 5 cm from the anus) was sectioned, and the intestinal contents were squeezed out and collected in sterile cryovials. All tissue samples were immediately frozen in liquid nitrogen after collection and then transferred to a −80 °C ultra-low temperature freezer for long-term storage. The crude protein, crude fat, crude ash, and moisture contents of the diet were determined using standard methods (AOAC, 1995).

All experimental operations and animal welfare protocols were strictly implemented under the approval of the Animal Ethics Committee of Yangtze River Fisheries Research Institute, Chinese Academy of Fishery Sciences (Approval No.: YFI2026066, Date: 20 January 2026), and complied with the ARRIVE guidelines and national laboratory animal welfare regulations.

The contents of serum alanine aminotransferase (ALT), aspartate aminotransferase (AST), alkaline phosphatase (ALP), triglycerides (TG), high-density lipoprotein cholesterol (HDL-C), low-density lipoprotein cholesterol (LDL-C), total cholesterol (TC), total protein (TP), and albumin (ALB) in the experimental fish were measured using a BS-460 fully automatic serum biochemical analyzer (Shenzhen Mindray Bio-Medical Electronics Co., Ltd., Shenzhen, China) and commercial reagent kits.

The contents of blood E2, T, cortisol, potassium ions, and chloride ions in the experimental fish were entrusted to Jingzhou Central Hospital in Hubei Province for measurement. The contents of blood E2 and T were measured using the chemiluminescence method.

Indicators such as complement protein 3 (C3), complement protein 4 (C4), immunoglobulin M (IgM), lipase (LPS), fatty acid synthase (FAS), lipoprotein lipase, acetyl-CoA carboxylase, and intestinal microbiota were entrusted to Nuomin Keda (Wuhan) Biotechnology Co., Ltd. (Wuhan, China) for measurement.

### 2.4. 16S rRNA Gene Sequencing and Bioinformatics Analysis

Total genomic DNA of intestinal content samples was extracted using a laboratory-prepared SDS method. The brief procedure was as follows: approximately 0.3 g of sample was placed in a grinding tube containing grinding beads, and 700 μL of SDS lysis buffer was added, followed by grinding for 3 min; incubation at 65 °C for 10 min, followed by centrifugation at 12,000 rpm for 5 min; the supernatant was collected, and an equal volume (approximately 600 μL) of Binding Buffer was added; after mixing, centrifugation was performed at 12,000 rpm for 5 min (if precipitation occurred); 400 μL of supernatant was mixed with an equal volume of absolute ethanol and 15 μL of magnetic beads (thoroughly mixed), and transferred to a 96-well plate; the remaining supernatant (no more than 400 μL) was mixed with an equal volume of absolute ethanol and transferred to the corresponding wells of the same 96-well plate; washing twice with 800 μL of 75% ethanol; finally, DNA was eluted with 150 μL of TE buffer.

The V3-V4 hypervariable region of the 16S rRNA gene was targeted using primers 341F (sequence: CCTAYGGGRBGCASCAG) and 806R (sequence: GGACTACNNGGTATCTAAT). PCR amplification conditions were as follows: initial denaturation at 95 °C for 2 min; 30 cycles of denaturation at 95 °C for 20 s, annealing at 50 °C for 30 s, and extension at 72 °C for 30 s; final extension at 72 °C for 5 min. After PCR product purification, paired-end sequencing was performed on the MGI DNBSEQ-G99 platform.

Quality control and bioinformatics analysis were performed using QIIME2 software for quality filtering (removal of low-quality reads and chimeras); OTU/ASV clustering at 97% similarity; taxonomic annotation using the Silva database (v138); rarefaction curves to assess sequencing depth sufficiency; alpha diversity indices (Chao1, Shannon, Simpson, ACE, Pielou, PD_whole_tree) and beta diversity analyses (PCoA, NMDS) to evaluate differences in microbial community structure between groups; and differential abundance testing using LEfSe (LDA Effect Size) and Wilcoxon rank-sum tests. Gut microbiota diversity indices were compared between groups using independent samples *t*-tests or Mann–Whitney U tests (depending on data normality and variance homogeneity), with significance set at *p* < 0.05. Raw sequencing data have been submitted to the NCBI Sequence Read Archive (SRA) database, and the BioProject accession number will be finalized and made publicly available during the proof stage. Intestinal microbiota were entrusted to Nuomin Keda (Wuhan) Biotechnology Co., Ltd. (Wuhan, China) for measurement.

### 2.5. Data Analysis

All samples used in the experiment were independent of each other, and the experimental data were expressed as mean ± standard deviation (mean ± SD). SPSS 22.0 software was used to analyze the differences between the freshwater group and the seawater group using an independent samples *t*-test, and Levene’s test for equality of variances was employed to assess the homogeneity of variances between groups. Differences were considered significant at *p* < 0.05.

## 3. Results

### 3.1. Growth Performance

The differences in growth performance of the F_2_ generation of Chinese sturgeon between freshwater and seawater environments are presented in Table 2. Seawater acclimation affected the growth of Chinese sturgeon juveniles, with the body weight and body length showing a slight decreasing trend compared with the freshwater group. However, no significant difference was observed between the two groups (Table 2).

### 3.2. Serum Biochemical Indices

The differences in serum biochemical indicators of the F_2_ generation of Chinese sturgeon between freshwater and seawater environments are presented in Table 3. The alkaline phosphatase (ALP) level in the MF group was significantly lower than that in the MS group (*p* < 0.05). The triglyceride (TG) level in the MF group was significantly lower than that in the MS group (*p* < 0.05). Both the total cholesterol (TC) and low-density lipoprotein cholesterol (LDL-C) levels in the MF group were significantly reduced (*p* < 0.05). Additionally, the albumin II (ALBII) level in the MF group was significantly lower than that in the MS group (*p* < 0.05).

### 3.3. Serum Hormones and Electrolytes

The differences in serum hormone levels of the F_2_ generation of Chinese sturgeon between freshwater and seawater environments are presented in Table 4. The thyroid hormone level in the MS group was significantly lower than that in the MF group (*p* < 0.05). The chloride ion content in the MF group was significantly lower than that in the MS group (*p* < 0.05).

### 3.4. Lipid Metabolic Enzyme Activities in Gill and Liver

The differences in the activities of lipid metabolism enzymes in the liver and gills of the F_2_ generation of Chinese sturgeon between freshwater and seawater environments are presented in Table 5. The lipase activity in the gills of the MF group was significantly lower than that in the MS group (*p* < 0.05). The fatty acid synthase activity in the gills of the MF group was significantly lower than that in the MS group (*p* < 0.05). Conversely, the lipoprotein lipase activity in the gills of the MF group was significantly higher than that in the MS group (*p* < 0.05).

### 3.5. Splenic and Serum Immune Indices

The differences in immune indicators of the F_2_ generation of Chinese sturgeon between freshwater and seawater environments are presented in Table 6. The spleen C4 level in the MF group was significantly lower than that in the MS group (*p* < 0.05), while no significant differences were observed for other indicators among the groups (*p* > 0.05).

### 3.6. Intestinal Microbiota Alpha Diversity and Community Structure

The differences in the α-diversity of intestinal microbiota in the F_2_ generation of Chinese sturgeon between freshwater and seawater environments are presented in Table 7. The α-diversity indices (Chao1, Shannon, Simpson, Pielou, ACE, and PD_whole_tree) of the intestinal microbiota in the MS group were significantly higher than those in the MF group (*p* < 0.05).

**Figure 1:**
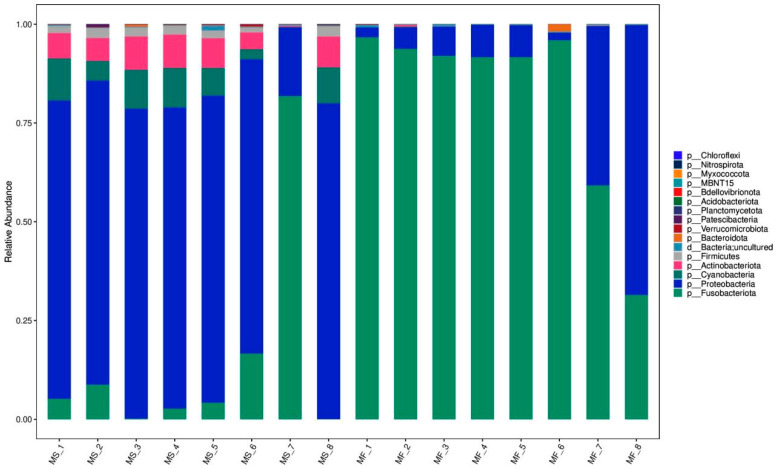
Community structure of the F_2_ generation intestinal microbiota in Chinese sturgeon at the phylum level under seawater and freshwater conditions. Note: The bars represent the average relative abundance of microbial communities in each group; MS denotes the seawater group, and MF denotes the freshwater group.

At the phylum level, there was a notable divergence in the intestinal microbiota structure between the two groups of Chinese sturgeon (Figure 1). In the MS group, *Proteobacteria* (69.6%) was the dominant phylum, followed by *Fusobacteriota* (14.9%). In contrast, *Fusobacteriota* dominated in the MF group (81.5%), with *Proteobacteria* accounting for only 17.8%.

**Figure 2:**
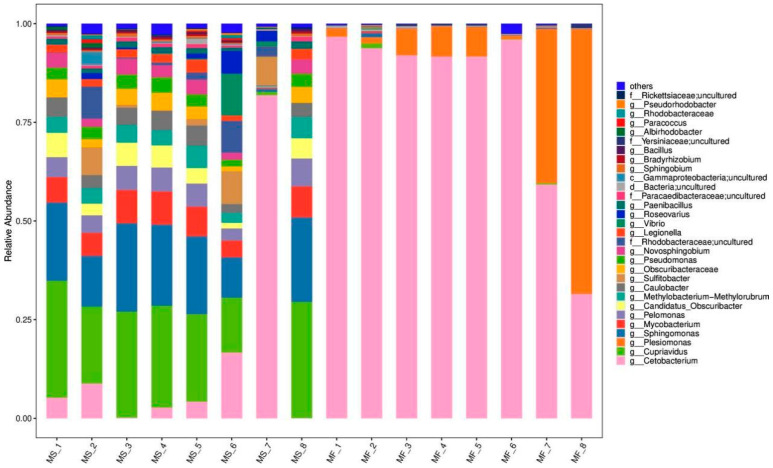
Community structure of the F_2_ generation gut microbiota in Chinese sturgeon at the genus level under seawater and freshwater conditions. Note: The bars represent the average relative abundance of microbial communities in each group; MS denotes the seawater group, and MF denotes the freshwater group.

At the genus level, the differences in microbial community composition between the two groups were even more pronounced (Figure 2). The MS group exhibited a more diverse array of dominant genera, including *Sphingomonas* (16.0%), *Cupriavidus* (21.0%), and *Cetobacterium* (14.9%). In contrast, *Cetobacterium* (81.5%) overwhelmingly dominated in the MF group, followed by *Plesiomonas* (16.5%).

## 4. Discussion

### 4.1. Effects of Salinity on Growth Performance

The present study found that the growth performance (body weight and length) of F_2_ Chinese sturgeon juveniles in the seawater acclimation group showed a declining trend, although this did not reach statistical significance. This observation can be explained from the perspectives of energy allocation, hormonal regulation, acclimation duration, and fish body size. At the level of energy allocation, salinity changes force fish to devote more energy to osmoregulation, thereby potentially reducing the energy available for somatic growth. Boeuf and Payan (2001) pointed out that 20% to over 50% of the total energy budget in fish is allocated to osmoregulation [1]. Consequently, when fish are exposed to high-salinity environments, a substantial proportion of energy is directed toward maintaining osmotic balance, resulting in a corresponding reduction in energy allocated to somatic growth. In Siberian sturgeon (*Acipenser baerii*) juveniles, individuals reared at the isosmotic point (≈250 mOsmol/kg) show better growth than those in hypo-osmotic freshwater conditions, indicating an energy trade-off between osmoregulation and growth in sturgeon [18]. Furthermore, a study on stellate sturgeon (*Acipenser stellatus*) juveniles found that as salinity and feeding frequency increased, major growth indices and feed efficiency showed upward trends, with the highest final body weight and length achieved at 12‰ salinity combined with six feedings per day [9]. ALP, ALT, and AST differed significantly among salinity treatment groups [9]. However, other studies have highlighted the influence of juvenile body size on salinity adaptation: no significant differences in mass-adjusted oxygen consumption rates were observed among different age groups of green sturgeon (*Acipenser medirostris*) in freshwater and seawater, suggesting that the energetic cost of osmoregulation may not be sufficiently high to fully account for the observed growth reduction [19]. The elevated serum lipid levels and altered enzyme activities observed in the seawater group are consistent with the view that energy may be preferentially channeled toward maintaining osmotic homeostasis. However, confirming whether this occurs at the direct expense of somatic growth would require quantitative measurements of whole-animal metabolic rate and energy budget allocation. From the perspective of hormonal regulation, the significant decrease in T3 levels observed in the seawater group in the present study may represent an intrinsic factor underlying the growth limitation. Environmental salinity significantly modulates the hypothalamic-pituitary-thyroid (HPT) axis in teleosts [20]. This series of findings provides strong evidence that T3 is a central regulator of growth in sturgeon. In the present study, the significant decrease in T3 without a significant change in T4 in the seawater group suggests that the high-salinity environment may primarily suppress the peripheral conversion efficiency of T4 to T3, rather than affecting thyroid hormone synthesis. The response of the thyroid axis to salinity is highly species-specific. For instance, while some species exhibit altered T3/T4 ratios under osmotic stress, in rainbow trout (*Oncorhynchus mykiss*) fingerlings, increasing water salinity significantly increased both serum T4 and T3 levels [21]. As a key regulator of growth and metabolism, the decline in T3 levels is one of the important reasons for the downward growth trend observed in the seawater group of the present study. With regard to acclimation duration and fish body size, Wang et al. investigated seawater acclimation of captive-bred Chinese sturgeon and Dabry’s sturgeon (*Acipenser dabryanus*) juveniles and found that Chinese sturgeon juveniles possess strong osmoregulatory and adaptive capacity in high-salinity environments. They can enhance gill filament Na^+^/K^+^-ATPase (NKA) activity by modulating hormone levels and stimulate increases in both the diameter and number of gill epithelial chloride cells, thereby achieving ion excretion and osmotic balance under high-salinity conditions. In contrast, Dabry’s sturgeon exhibited a narrower salinity tolerance range, and salinities exceeding a certain threshold caused irreversible damage to tissue structure [5]. These results confirm, on the one hand, that Chinese sturgeon juveniles possess the physiological basis for survival at salinities of 15 to 23.2 psu, while on the other hand indicating significant interspecies differences in salinity adaptation among sturgeon species. The present study employed a stepwise salinity increase protocol ranging from 15 to 23.2 psu with an acclimation period of 30 days. This duration may be insufficient for the seawater group to complete the full transition from the “stress adaptation phase” to the “metabolic compensation phase”—the fact that growth in the seawater group showed only a declining trend without reaching statistical significance may be related to this acclimation stage coinciding with a critical window of energy reallocation. Moreover, the non-significant elevation of cortisol further indicates that the stepwise acclimation protocol effectively avoided the strong growth-suppressive effects of acute stress. Wang et al. reported that serum cortisol levels in Chinese sturgeon juveniles increased significantly under high-salinity conditions, whereas no significant change was observed in Dabry’s sturgeon [5]. This result differs from the non-significant change in cortisol observed in the present study—the primary reason lies in the difference in acclimation protocols: Wang et al. employed a continuous salinity increase (acute acclimation) protocol, whereas the present study adopted a more gradual stepwise approach. Therefore, the absence of a significant cortisol increase is precisely an indicator of successful slow salinity adaptation in the present experiment. In summary, the results of this study suggest that F_2_ Chinese sturgeon juveniles grow more favorably under freshwater low-osmotic conditions; seawater acclimation induced a declining trend in growth performance without statistical significance. The individual developmental stage, acclimation duration, and acclimation protocol are important modulating factors influencing the salinity–growth relationship.

### 4.2. Effects of Salinity on Serum Biochemical Parameters

Serum biochemical parameters are sensitive indicators reflecting the nutritional metabolic status, liver health, and stress level in fish [22]. In the present study, no significant differences in ALT and AST were observed between the two salinity conditions. Mozanzadeh et al. demonstrated that dietary nutrient deficiency can affect plasma protein levels without necessarily indicating impaired hepatic synthetic function, suggesting that serum transaminase levels are regulated by multiple factors [23]. The stability of ALT and AST in the present study further indicates that the stepwise seawater acclimation did not cause substantial cellular damage to the liver of Chinese sturgeon. Chen et al. also confirmed in red tilapia that serum ALT and AST levels exhibited regular patterns across different salinity treatments, and stable transaminase levels are an important hallmark of successful salinity adaptation in fish [24]. The normal ALT and AST levels in the seawater group suggest that the salinity range used in this study (15–23.2 psu) remains within the physiological tolerance limit of Chinese sturgeon juveniles. ALP is a key enzyme involved in substance metabolism and stress response. In the present study, the freshwater group exhibited significantly lower ALP levels than the seawater group. In stellate sturgeon juveniles, ALP, ALT, and AST levels all differed significantly among salinity and feeding frequency treatment groups, indicating that ALP is sensitive to salinity changes [9]. Under freshwater culture conditions, the hepatic and intestinal physiological states of Chinese sturgeon were more stable and were not subjected to additional disturbance from salinity stress. The seawater group showed significantly elevated TG, TC, and LDL-C levels. Rizzatti et al. systematically reviewed the close association between Proteobacteria and host metabolism [25]. In Nile tilapia under salinity stress, the expression of glycolipid metabolism-related genes (e.g., LPL) changed significantly, confirming the profound regulatory role of salinity on fish lipid metabolism [26]. The elevated TG, TC, and LDL-C levels in the seawater group of the present study suggest that, during adaptation to the high-salinity environment, Chinese sturgeon tends to mobilize lipid reserves to meet the energy demands of osmoregulation, whereas the freshwater environment maintains a lower lipid metabolic burden. Notably, the significant increase in TG in the seawater group coincided with a declining growth trend; this seemingly paradoxical phenomenon precisely indicates that lipid mobilization induced by the high-salinity environment is a stress response rather than a growth-promoting mechanism: lipids are recruited to pay the energetic cost of osmoregulation, but the overall shift in energy allocation strategy reduces the energy remaining for somatic growth. Therefore, despite elevated blood lipid levels, growth does not benefit. The freshwater group showed significantly lower ALBII levels than the seawater group, whereas no significant difference in TP was observed. In tilapia, salinity changes can significantly affect serum biochemical indices including hormone and protein indicators [24]. This reflects changes in protein turnover rate rather than substantial impairment of hepatic synthetic function. In fish, low-salinity environments maintain a lower protein metabolic rate, which helps optimize energy use efficiency.

### 4.3. Effects of Salinity on Serum Hormones and Electrolytes

One of the most significant findings of the present study was the markedly reduced T3 level in the seawater group, whereas no significant differences in TSH and T4 were observed between the two groups. Research on thyroid hormone levels across different developmental stages of lake sturgeon (*Acipenser fulvescens*) revealed that the tissue distribution and content of T3 and T4 exhibit significant developmental stage specificity [27]. In lake sturgeon, application of 3 ppm T3 at fertilization significantly improved growth and survival during the first month post-hatch, accompanied by synchronous upregulation of appetite-regulating genes and somatic growth gene (insulin-like growth factor 1) mRNA abundance [27]. This specific pattern, decreased T3 with unchanged T4, is consistent with the hypothesis that high-salinity conditions may influence thyroid hormone dynamics, potentially through effects on peripheral T4-to-T3 conversion or alterations in thyroid hormone clearance rates. Distinguishing among these possibilities would require direct measurements of deiodinase activities and hormone turnover kinetics in future studies. During the smoltification of Atlantic salmon, both T3 and T4 levels decreased significantly after seawater transfer, which is consistent with the T3 variation pattern observed in the present study. Salinity changes modulate fish hormone secretion through the hypothalamus–pituitary–thyroid axis, and high-salinity environments inhibit T3 synthesis, thereby reducing metabolic adaptability and growth potential [28]. As a central regulator of fish metabolism and growth, the decline in T3 levels is one of the key reasons for the downward growth trend observed in the seawater group. Cortisol is a core indicator reflecting the acute stress status in fish [29]. In the present study, no significant difference in cortisol was found between the freshwater and seawater groups. Wang et al. reported that serum cortisol levels in Chinese sturgeon juveniles increased significantly under high-salinity conditions; this discrepancy arises from differences in the acclimation protocol [5]. Chang et al. reviewed that in fish, cortisol functions both as a glucocorticoid and a mineralocorticoid, supporting salinity adaptation by modulating energy pathways that control glucose availability [28]. Studies on Atlantic salmon have shown that salinity changes can induce molecular-level stress and immune responses even months after smoltification is complete; however, changes in acute stress indicators such as cortisol are time-dependent, with plasma stress markers elevated at 20 h post-transfer and partially recovered after 2 weeks [30]. Environmental factors such as temperature and salinity significantly affect serum immune factor activity in teleost fish, often exhibiting significant synergistic effects on immune parameters like complement components [31]. In goldfish, plasma cortisol, glucose, and Na^+^ concentrations all increased after 21 days of exposure to critical salinity, indicating that high-salinity environments can induce significant stress [32]. The absence of cortisol elevation in the present study indicates that the gentle stepwise acclimation effectively avoided acute stress, which constitutes an important physiological basis for the seawater group to complete adaptation with 100% survival while experiencing certain growth limitation. Cl^−^ content was significantly lower in the freshwater group than in the seawater group, whereas no significant differences in K^+^, Na^+^, or Ca^2+^ were observed between the two groups. Cl^−^ is an important indicator reflecting osmoregulatory strategy [33]; the seawater acclimation group actively absorbs and retains Cl^−^ to maintain osmotic balance, whereas the freshwater group minimizes ion loss through gill and renal transport systems. Although Na^+^ showed no significant difference in the present study, this may be attributed to the establishment of a new ionic homeostasis after 30 days of acclimation, whereas the persistent difference in Cl^−^ reflects a more sensitive indicator of ionic regulation.

### 4.4. Regulation of Lipid Metabolic Enzyme Activities in Gill and Liver Tissues by Salinity

The gill is the most critical osmoregulatory organ in sturgeon [34]. In the present study, both gill LPS and FAS activities were significantly lower in the freshwater group than in the seawater group, whereas LPL activity was significantly higher in the freshwater group. This enzymatic activity profile indicates that under freshwater conditions, Chinese sturgeon maintains osmotic balance with relatively low energy expenditure by suppressing de novo lipid synthesis (evidenced by inhibited FAS and LPS activities) while simultaneously enhancing lipid catabolism (evidenced by elevated LPL activity). The effects of salinity stress on fish lipid metabolism involve complex molecular regulatory networks [35]. In Nile tilapia, hepatic LPL expression in the salinity stress group was significantly higher than in the freshwater group, confirming the pronounced regulatory role of salinity stress on lipid metabolism-related gene expression in fish [26]. Li et al. demonstrated that ambient salinity and dietary fatty acids can interactively regulate hepatic fatty acid composition and desaturase expression in rabbitfish (*Siganus canaliculatus*), indicating that salinity can reshape lipid metabolic patterns by modulating the expression of lipid metabolism-related genes [36]. Notably, in black porgy (*Acanthopagrus schlegelii*) juveniles, both gill and liver tissues exhibited significant gene expression changes under hypo-osmotic (0.5 psu), iso-osmotic (12 psu), and hyper-osmotic (35 psu) salinities, revealing a synergistic regulatory mechanism between gill and liver during salinity adaptation [37]. In contrast, no significant differences in any hepatic lipid metabolic enzyme activities were observed between the freshwater and seawater groups in the present study. This result is consistent with the findings in black porgy, indicating that although both gill and liver participate in salinity adaptation, their respective response patterns and regulatory mechanisms exhibit marked tissue specificity [37]. This reveals that salinity regulation of lipid metabolism in Chinese sturgeon is highly tissue-specific: the gill, as the tissue in direct contact with the external environment, displays greater metabolic plasticity and response sensitivity to salinity fluctuations [38], whereas the liver, as the core organ maintaining systemic metabolic homeostasis, is relatively less affected by salinity changes and maintains relatively stable enzyme activity levels across a broad salinity range [39]. This tissue-specific regulatory characteristic is of great significance for understanding the functional division of different organs in osmotic adaptation. Notably, in the present study, serum TG, TC, and LDL-C were significantly elevated in the seawater group, yet hepatic LPS and FAS activities did not change correspondingly. This dissociation elevated serum lipid metabolites without concurrent changes in hepatic synthetic enzyme activities. The dissociation between elevated serum lipid metabolites and unaltered hepatic synthetic enzyme activities raises the possibility that the observed hyperlipidemia may not stem from enhanced hepatic de novo lipogenesis. A plausible alternative is that lipid redistribution among tissues—particularly peripheral osmoregulatory organs such as the gill—may contribute to this pattern, as has been suggested in other euryhaline species [40]. Direct tissue-specific lipidomic profiling would be required to test this hypothesis. Previous studies have confirmed that euryhaline fish can undergo inter-tissue lipid redistribution during seawater adaptation [40]. Therefore, the lipid metabolic adaptation strategy of Chinese sturgeon to high-salinity environments may involve “inter-tissue redistribution” rather than simple upregulation of hepatic synthesis.

### 4.5. Regulation of Splenic Immune Function by Salinity

Complement C3, C4, and IgM are core effector molecules of the innate immune system in fish [41]. The present study found that splenic complement C4 levels were significantly higher in the seawater group than in the freshwater group, whereas splenic C3, IgM, and all serum immune indicators showed no significant differences between the two groups. The elevated splenic C4 level in the seawater group points to a localized immunomodulatory response in the spleen during salinity challenge. Whether this represents a compensatory mechanism to preserve immune homeostasis, or reflects other aspects of immune system adjustment, remains to be determined. Functional immune assays—such as phagocytic activity or respiratory burst—would be needed to further characterize this response. The significant elevation of splenic C4 in the seawater group suggests that salinity stress activates a local immunocompensatory mechanism in the spleen, a peripheral immune organ, to maintain immune homeostasis under osmotic challenge [42]. Suo et al. reported in Nile tilapia that chronic hyperosmotic stress can induce splenomegaly, reduce coagulation function, enhance phagocytic activity, and downregulate the complement pathway at the transcriptional level in the spleen. Moreover, the spleen was identified as a more sensitive immune organ than the head kidney under chronic salinity stress [43]. However, this pattern differs from the elevated splenic C4 observed in the present study; this may reflect species specificity: in sturgeon, a more phylogenetically primitive fish group, the salinity-induced splenic immune response may manifest as compensatory upregulation of complement C4 rather than overall downregulation of the complement pathway, representing a unique immune adaptation strategy of sturgeon relative to more derived teleosts. Suo et al. also noted that the complement system comprises a large number of plasma proteins and plays a central role in innate immunity, with its regulation closely linked to pro-inflammatory gene expression [43]. Wang et al. investigated splenic immune regulation in koi carp (*Cyprinus carpio Koi*) following Aeromonas hydrophila infection and found that complement-related genes such as *c2* and *c3* were downregulated, further confirming the central role of complement system regulation in splenic immune responses in fish [44]. In common carp (*Cyprinus carpio*), salinity stress involves coordinated responses of the nervous, endocrine, and immune systems, and hyperosmotic stress exerts significant transcriptional regulation on immune organs such as the spleen [45]. In Atlantic salmon, salinity changes can induce molecular-level stress and immune responses; cytokine signals (e.g., il1b) and antimicrobial defense-related gene expression changes were still detectable months after smoltification upon seawater transfer [30]. Meanwhile, the invariance of serum immune indicators suggests that the salinity-induced immune response is primarily confined to local immune organs rather than triggering systemic immune fluctuations [46]. In L. capito under high-salinity stress, the numbers of white blood cells, lymphocytes, neutrophils, and erythrocytes in the spleen changed significantly, and the expression of *HSP70*, *HSP90*, *CAT*, *SOD*, and *GPX1* genes was markedly upregulated. GO enrichment analysis showed that immune system processes, multicellular organismal processes, and biological regulation were the most significantly enriched biological processes, while KEGG analysis revealed significant enrichment of lipolysis regulation, FoxO signaling pathway, hematopoietic cell lineage signaling pathway, and HIF-1 signaling pathway [47]. This transcriptomic evidence further supports the central immunoregulatory function of the spleen during salinity adaptation. Taken together, the elevation of splenic C4 in the present study may reflect that Chinese sturgeon maintains immune homeostasis under high-salinity conditions through local regulation of the complement system, and the cost of this immune adaptation may indirectly affect growth performance through alterations in energy allocation.

### 4.6. Effects of Salinity on Gut Microbial Community Structure and Diversity

The gut microbial community is closely associated with host nutritional metabolism, immune regulation [48], and osmotic adaptation [49]. The present study found that all α-diversity indices (Chao1, Shannon, Simpson, and ACE) of the gut microbiota were significantly higher in the seawater group than in the freshwater group. Leng et al. reported in Chinese sturgeon that seawater acclimation significantly altered gut microbial composition and serum metabolite profiles. 16S rRNA analysis revealed significant enrichment of Bacillaceae, Ruminococcaceae, Eimeria praecox, and Rhodobacteraceae under high-osmotic salinity, while broad-targeted metabolomics identified 123 upregulated and 30 downregulated metabolites, with differential metabolites primarily involved in carbohydrate metabolism [17]. That study also found significant correlations between metabolite changes and bacterial community shifts, particularly involving Ruminococcaceae and Prevotellaceae [17]. These findings indicate that ambient salinity can modulate gut microbial composition and serum metabolites, and that changes in the abundance of specific gut taxa can influence carbohydrate metabolism, thereby enhancing carbohydrate utilization to meet the energy demands during seawater adaptation; this is highly consistent with the present observation of a shift toward a Proteobacteria-dominated gut community structure in the seawater group [17,50,51]. Rizzatti et al. systematically reviewed the common commensal characteristics of Proteobacteria in the host environment [25]. Furthermore, the response of gut microbiota to salinity is a universal phenomenon. In Qinghai Lake naked carp (*Gymnocypris przewalskii*), different salinity treatments (0, 5, 10, and 15 psu) significantly altered gut microbial community structure, confirming the systematic impact of salinity on the gut microecology of fish [25]. In mandarin fish (*Siniperca chuatsi*), hyperosmotic stress induced by high salinity significantly altered the intestinal microbial composition and reduced microbial diversity, while also affecting transcriptomic profiles and immune function in the gut [11]. In Nile tilapia (*Oreochromis niloticus*), acute high-salinity stress significantly reshaped the gut microbial community structure, leading to marked changes in species composition and alpha diversity, as revealed by metagenomic analysis [50]. In spotted sea bass (*Lateolabrax maculatus*), salinity regulates osmotic adaptation through sterol metabolism and fatty acid catabolism (e.g., *PPAR* signaling pathway) [52]. Collectively, these studies demonstrate that salinity comprehensively reshapes the gut microecology by altering intestinal osmotic pressure, mucosal immune status, and host metabolic characteristics. In the present study, the seawater group exhibited higher gut microbial α-diversity and a more complex community structure; The host needs to invest more metabolic energy to sustain a more diverse gut microbial equilibrium [53], which may partially explain the growth decline observed in the seawater group. More importantly, Leng et al. found that serum metabolite changes during seawater adaptation in Chinese sturgeon were significantly correlated with gut microbiota composition, suggesting that the gut microbiota–host metabolic axis plays a key role in salinity adaptation [17]. These findings provide important metabolomic and microbiomic evidence for understanding the seawater adaptation strategy of Chinese sturgeon. The observed correlations between gut microbial community shifts and serum metabolite changes, as also reported in Chinese sturgeon by Leng et al. [17], suggest an association between microbiota composition and host metabolic status during seawater acclimation. Nonetheless, correlation does not establish causation. Future functional studies—including fecal microbiota transplantation, metabolomic profiling, or transcriptomic analysis—are needed to determine whether and how gut microbiota remodeling directly influences host energy metabolism and growth under salinity stress.

### 4.7. Integrated Analysis of Gut Microbiota and Host Physiological Responses

Simultaneous assessment of gut microbiota, serum biochemical parameters, thyroid hormones, lipid metabolism enzymes, and spleen immune indicators is important for elucidating salinity adaptation mechanisms in F_2_-generation Acipenser sinensis. Single physiological indicators mainly reflect phenotypic responses under osmotic stress, but are insufficient to comprehensively reveal potential microbiota-associated regulatory processe [48,54] The intestine serves as a key interface between external salinity conditions and host physiological status. Previous studies suggest that microbial metabolites, such as short-chain fatty acids and bile acid derivatives, may enter systemic circulation and be associated with lipid metabolism, ion homeostasis, and immune signaling processes [54,55]. Therefore, integrating gut microbiota profiles with multi-dimensional physiological parameters may provide a more holistic perspective on the relationship between salinity variation and host physiological responses [48].

The present results indicate that differences in gut microbiota composition between the two groups are generally consistent with variations in lipid metabolism, thyroid hormones, chloride levels, and spleen immune indicators. Regarding lipid metabolism, a higher relative abundance of Proteobacteria in the seawater group may be associated with enhanced lipid metabolic activity, whereas the dominance of Cetobacterium in the freshwater group appears to correspond to lower serum lipid levels and variations in gill lipid metabolism enzyme activity [55,56]. In terms of ion homeostasis, salinity changes were accompanied by alterations in gut microbial composition, which may be linked to changes in intestinal epithelial chloride transport processes, resulting in differences in serum chloride levels [36,42]. Regarding thyroid regulation, microbial metabolites may be associated with peripheral thyroid hormone conversion processes, and the observed reduction in T3 levels in seawater individuals suggests that this process may be influenced by multiple factors [42,55]. In terms of immune responses, changes in microbial diversity in the seawater group may be associated with intestinal mucosal immune activation, which is further linked to variations in splenic complement C4 levels, whereas systemic serum immune parameters remained relatively stable [42,54]. In addition, tissue-specific differences were observed in lipid metabolism responses, with more pronounced changes in gill tissue, while hepatic lipid metabolism enzymes showed no significant differences between groups [36].

Overall, salinity variation may influence host physiological status through modulation of gut microbial composition and activity, potentially contributing to multi-level associations with lipid metabolism, endocrine regulation, ion homeostasis, and immune responses. Integrated analysis of gut microbiota and host physiological parameters provides a useful framework for understanding systemic adaptive responses in juvenile Acipenser sinensis under salinity stress [48].

## 5. Conclusions

The 30-day seawater acclimation (15–23.2 psu) did not exert a significant effect on the growth of F_2_ Chinese sturgeon juveniles, but induced multiple adaptive changes at the physiological level. In the seawater group, serum triglycerides, total cholesterol, low-density lipoprotein cholesterol, alkaline phosphatase, and albumin II levels were significantly elevated, whereas alanine aminotransferase and aspartate aminotransferase showed no intergroup differences, indicating that the seawater environment promoted lipid mobilization without causing hepatic damage. Serum triiodothyronine was significantly reduced, and chloride was significantly elevated, while cortisol remained unchanged, suggesting that seawater suppressed thyroid hormone secretion without triggering an acute stress response. Gill lipase and fatty acid synthase activities were significantly lower in the freshwater group, whereas lipoprotein lipase activity was higher; no intergroup differences in hepatic enzyme activities were observed, indicating that freshwater favors lipid catabolism in gill tissue and that regulation exhibits tissue specificity. Splenic complement C4 content was significantly higher in the seawater group, whereas complement C3 and immunoglobulin M showed no differences, reflecting local immunocompensation in the spleen rather than systemic immune fluctuation. Gut microbial α-diversity was significantly higher in the seawater group, with the community dominated by *Proteobacteria* (69.6%) at the phylum level, compared with *Fusobacteriota* (81.5%) in the freshwater group.

In summary, the freshwater environment is more conducive to maintaining a lower lipid metabolic burden and higher thyroid hormone levels, whereas the seawater environment induces increased gut microbial diversity and local splenic immunocompensation. In practical aquaculture, appropriate salinity conditions can be selected according to specific needs, with attention paid to changes in the aforementioned physiological indicators, to optimize the health management of Chinese sturgeon.

Nevertheless, one important limitation of this trial should be acknowledged: each salinity treatment was only arranged with a single rearing tank. Even though multiple individuals were sampled within each group, this design cannot fully separate salinity-induced physiological alterations from tank-specific environmental interference, and pseudoreplication cannot be completely ruled out. Accordingly, all physiological responses observed in the present study should be regarded as exploratory preliminary evidence rather than definitive salinity-specific conclusions. It is worth mentioning that all tanks were controlled under identical environmental parameters except salinity, which partly reduces irrelevant confounding factors and guarantees the basic comparability of the two groups. Future experiments with multiple independent replicate tanks per treatment are required to further validate the salinity-adaptive phenotypes of F_2_ Chinese sturgeon described in this work.

## Figures and Tables

**Table 1:** Composition and nutritional levels of basic diet.

Ingredient	Content/%	Proximate Composition	Content/%
Peruvian fish meal	37.00	Moisture	10.55
Defatted soybean meal	12.00	Crude protein	42.64
Casein	8.00	Crude fat	12.85
Gelatin	2.00	Crude ash	8.53
Fish oil	9.00		
Arachidonic-enrich oil	1.00		
Flour	21.00		
Choline chloride	0.20		
Ca(H_2_PO_4_)_2_	1.00		
Premix *	3.00		
Yeast	1.00		
Micro-cellose	2.80		

**Note:** * Contained the following vitamin premix per kg of diet (mg/kg diet): V_B1_ 50 mg, V_B2_ 200 mg, V_B6_ 50 mg, V_B12_ 20 mg, folic acid 15 mg, pantothenate 400 mg, inositol 1500 mg, *D*-biotin (2%) 5 mg, niacin 750 mg, V_A_ 2.5 mg, V_E_ (50%) 160 mg, V_D3_ 2 mg, V_K_ 20 mg. * Contained the following mineral premix per kg of diet (mg/kg diet): Ca(H_2_PO_4_)_2_ 1800 mg, KH_2_PO_4_ 1350 mg, NaCl 500 mg, MgSO_4_·7H_2_O 750 mg, NaH_2_PO_4_·2H_2_O 650 mg, KI 1.5 mg, COSO_4_·6H_2_O 2.5 mg, CuSO_4_·5H_2_O 15 mg, ZnSO_4_·7H_2_O 350 mg, FeSO_4_·7H_2_O 1250 mg, MnSO_4_·4H_2_O 80 mg, Na_2_SeO_3_ 6.00 mg.

**Table 2:** Effects on the growth of F_2_ generation Chinese sturgeon (*Acipenser sinensis*) under seawater and freshwater conditions.

Items	Group	
	MS	MF
Initial body weight (IBW, g)	2596 ± 747.7	2531 ± 339.9
Final body weight (FBW, g)	2591 ± 811.1	3057 ± 522.1
Initial body length (IBL, cm)	67.07 ± 5.78	67.93 ± 3.32
Final body length (FBL, cm)	68.86 ± 6.62	72.93 ± 3.61

**Note:** The sample size for all indicators is n = 14.

**Table 3:** Effects on serum biochemical indices in F_2_ generation Chinese sturgeon (*Acipenser sinensis*) under seawater and freshwater conditions.

Items	Group	
	MS	MF
Glucose (Glu-G, mmol/L)	1.64 ± 1.54	2.06 ± 1.06
Alanine aminotransferase (ALT, U/L)	96.8 ± 111.36	201.74 ± 112.11
Aspartate aminotransferase (AST, U/L)	255.55 ± 169.81	307.4 ± 144.36
Alkaline phosphatase (ALP, U/L)	94.06 ± 34.84 ^a^	63.4 ± 13.82 ^b^
Triglycerides (TG, mmol/L)	7.68 ± 5.08 ^a^	1.18 ± 0.41 ^b^
Low-density lipoprotein cholesterol (LDL-C, mmol/L)	2.31 ± 1.03 ^a^	0.74 ± 0.32 ^b^
High-density lipoprotein cholesterol (HDL-C, mmol/L)	0.32 ± 0.12	0.24 ± 0.03
Total cholesterol (TC, mmol/L)	2.74 ± 1.28 ^a^	1.07 ± 0.26 ^b^
Albumin II fraction (ALB-II, g/L)	7.99 ± 1.21 ^a^	5.28 ± 1.96 ^b^
Total protein II fraction (TP-II, g/L)	17.28 ± 7.36	15.54 ± 5.10

**Note**: Different lowercase letters in the same column indicate significant differences between groups (*p* < 0.05). The sample size for all indicators is n = 8.

**Table 4:** Effects on serum hormones in F_2_ generation Chinese sturgeon (*Acipenser sinensis*) in freshwater and seawater environments.

Items	Group	
	MS	MF
Cortisol (COR, ng/mL)	14.31 ± 3.99	13.56 ± 4.57
Testosterone (TESTO, ng/mL)	0.63 ± 0.18	0.71 ± 0.36
K^+^	3.13 ± 0.51	3.15 ± 0.30
Na^+^	141.85 ± 5.23	134.80 ± 5.15
Cl^−^	125.85 ± 5.93 ^a^	117.86 ± 2.31 ^b^
Ca^2+^	1.98 ± 0.08	1.88 ± 0.10
Thyroid-stimulating hormone (TSH, µIU/mL)	0.015 ± 0.010	0.016 ± 0.005
Triiodothyronine (T3, ng/mL)	0.29 ± 0.13 ^b^	0.55 ± 0.14 ^a^
Thyroxine (T4, ng/mL)	0.26 ± 0.11	0.28 ± 0.03

**Note**: Different lowercase letters in the same column indicate significant differences between groups (*p* < 0.05). Testosterone (TESTO): sample size n = 5; other indicators: sample size n = 8.

**Table 5:** Effects on hepatic and branchial lipid metabolic enzyme activities in F_2_ generation Chinese sturgeon (*Acipenser sinensis*) under seawater and freshwater conditions.

Items	Group	
	MS	MF
Liver		
Lipase (LPS, U/g tissue)	0.89 ± 0.12	1.19 ± 0.44
Fatty acid synthase (FAS, U/g tissue)	313.64 ± 271.31	315.52 ± 133.25
Acetyl-CoA carboxylase (ACC, U/g tissue)	761.83 ± 367.70	858.79 ± 107.08
Lipoprotein lipase (LPL, U/g tissue)	5.45 ± 0.20	5.76 ± 0.68
Gill		
Lipase (LPS, U/g tissue)	0.69 ± 0.20 ^a^	0.49 ± 0.15 ^b^
Fatty acid synthase (FAS, U/g tissue)	443.47 ± 229.51 ^a^	236.93 ± 106.73 ^b^
Acetyl-CoA carboxylase (ACC, U/g tissue)	1100.58 ± 239.61	1193.79 ± 270.15
Lipoprotein lipase (LPL, U/g tissue)	5.51 ± 0.23 ^a^	6.52 ± 0.66 ^b^

**Note:** Different lowercase letters in the same column indicate significant differences between groups (*p* < 0.05). The sample size for all indicators is n = 8.

**Table 6:** Effects on immune indicators of F_2_ generation Chinese sturgeon (*Acipenser sinensis*) under seawater and freshwater conditions.

Items	Group	
	MS	MF
Splenic		
Complement component 3 (C3, μg/g tissue)	1273.14 ± 289.95	1377.95 ± 336.90
Complement component 4 (C4, μg/g tissue)	2238.71 ± 560.62 ^a^	1495.29 ± 447.18 ^b^
Immunoglobulin M (IgM, ng/g tissue)	38,017.93 ± 6895.59	34,126.10 ± 4433.60
Serum		
Complement component 3 (C3, μg/g tissue)	109.21 ± 14.46	93.92 ± 15.31
Complement component 4 (C4, μg/g tissue)	142.19 ± 32.05	177.25 ± 42.06
Immunoglobulin M (IgM, ng/g tissue)	3111.78 ± 250.04	3040.19 ± 238.20

**Note:** Different lowercase letters in the same column indicate significant differences between groups (*p* < 0.05). The sample size for all indicators is n = 8.

**Table 7:** Effects on intestinal microbiota α-diversity in F_2_ generation Chinese sturgeon (*Acipenser sinensis*) under seawater and freshwater conditions.

Items	Group	
	MS	MF
Sequencing Depth Index		
Good’s Coverage Index	0.99 ± 0.00	0.99 ± 0.00
Bacterial Community Abundance Index		
chao1 Index	143.97 ± 26.09 ^a^	71.63 ± 8.82 ^b^
ACE Index	143.71 ± 24.48 ^a^	74.81 ± 8.33 ^b^
Bacterial Community Diversity Index		
Shannon Index	2.44 ± 0.65 ^a^	0.45 ± 0.22 ^b^
Simpson Index	0.81 ± 0.20 ^a^	0.21 ± 0.17 ^b^
Pielou Index	0.50 ± 0.13 ^a^	0.11 ± 0.05 ^b^
Phylogenetic Diversity Index		
PD-Whole-Tree Index	17.56 ± 1.88 ^a^	13.17 ± 1.37 ^b^

**Note:** Different lowercase letters in the same column indicate significant differences between groups (*p* < 0.05). The sample size for all indicators is n = 8.

## Data Availability

Data analyzed in the present study are included in the article.

## References

[1] Boeuf G., Payan P. (2001). How should salinity influence fish growth?. Comp. Biochem. Physiol. Part C Toxicol. Pharmacol..

[2] Xu L., Zhou L., Wei Q. (2023). Stock status and conservation dilemma of species of Acipenseriformes in the Yangtze River and relevant suggestions. J. Fish. China.

[3] Zhou Q., Du H., Wang J., Shao Y., Yan Z.G. (2024). Distribution characteristics of Chinese sturgeon in the Yangtze River based on environmental DNA. J. Environ. Eng. Technol..

[4] Jiang W., Du H.J., Chen P., Yang J., Li Z.Y. (2024). Process, difficulties and directions of species conservation of Chinese sturgeon. Acta Hydrobiol. Sin..

[5] Wang P.Y., Leng X.Q., Ren F.X., Zhong J., Cheng P.L., Zhang L.N., Qiao X.M., Du H. (2025). Comparative analysis of osmoregulatory capacity in freshwater-cultured juveniles of *Acipenser sinensis* and *Acipenser dabryanus*. J. Fish. China.

[6] Qin S.Z., Leng X.Q., Luo J., Du H., Liu Z.G., Qiao X.M., Xiong W., Wei Q.W. (2021). Adaptive adjustment of osmotic organ structure of juvenile Chinese sturgeon under seawater conditions. Prog. Fish. Sci..

[7] Iwama G.K., Takemura A. (1994). Osmotic Regulation and Growth in Fish. Fish Physiol. Biochem..

[8] Tseng Y.C., Hwang P.P. (2008). Some insights into energy metabolism for osmoregulation in fish. Comp. Biochem. Physiol. Part C Toxicol. Pharmacol..

[9] Ahmadi S.M., Vahabzadeh Roudsari H., Khara H., Pajand Z.O., Sayad Borani M. (2024). Optimum Growth, Enzymatic and Biochemical Reactions of Stellate Sturgeon (*Acipenser stellatus*) Juveniles in Response to the Feeding Frequency and Exposure to Environmental Salinity. Casp. J. Environ. Sci..

[10] Jiang B.J., Huang R.S., Tao Y.F., Lu S.Q., Hua J.X., Li Y., Dong Y.L., Xu P., Qiang J. (2025). Multi-omics and biochemical analyses provide insights into hepatic glucolipid metabolism in red tilapia (*Oreochromis* spp.) under salinity stress. Aquaculture.

[11] Xiong S., Liu Y., Zhu J., Li J., Shen Y., Wang G., Wu H. (2022). Effects of Hyperosmotic Stress on the Intestinal Microbiota, Transcriptome, and Immune Function of Mandarin Fish (*Siniperca chuatsi*). Aquaculture.

[12] McKenzie D.J., Cataldi E., Romano P., Owen S.F., Taylor E.W., Bronzi P. (2001). Effects of acclimation to brackish water on the growth, respiratory metabolism, and swimming performance of young-of-the-year Adriatic sturgeon (*Acipenser naccarii*). Can. J. Fish. Aquat. Sci..

[13] Likongwe J.S., Stecko T.D., Stauffer J.R., Carline R.F. (1996). Combined effects of water temperature and salinity on growth and feed utilization of juvenile Nile tilapia Oreochromis niloticus (*Linnaeus*). Aquaculture.

[14] Su M.L., Feng Z.Q., Zhong Y.L., Ye Z., Zhang J. (2025). Insights into salinity effect on growth of the spotted scat (*Scatophagus argus*): Exploring the optimum salinity for its culture. Aquac. Rep..

[15] Wang Y., Guo Q., Zhao H., Liu H., Lu W. (2015). Larval development and salinity tolerance of Japanese flounder (*Paralichthys olivaceus*) fromhatching to juvenile settlement. Aquac. Res..

[16] Liu Y., Tian J., Li S., Zhu T., Du J., Hongmei S. (2026). Effects of chronic salinity stress on growth performance, physiological response and intestinal microbiota of largemouth bass (*Micropterus salmoides*). Comp. Biochem. Physiol. Part A Mol. Integr. Physiol..

[17] Leng X.Q., Luo J., Zhong J., Wu J.P., Qiao X.M., Xiong W., Liu Z.G., Du H. (2025). Integrated gut microbiome and serum metabolomics analysis to reveal seawater adaptation strategy in Chinese sturgeon. Aquacult. Rep..

[18] Rodríguez A., Gallardo M.A., Gisbert E., Castelló-Orvay F., Santilari S., Ibarz A., Sánchez J. (2002). Osmoregulation in juvenile Siberian sturgeon (*Acipenser baerii*). Fish Physiol. Biochem..

[19] Allen P.J., Cech J.J. (2007). Age/size eftects on juvenile green sturgeon, *Acipenser medirostris*, oxygen consumption, growth, and osmoregulation in saline environments. Environ. Biol. Fishes.

[20] Ruiz-Jarabo I., Martos-Sitcha J.A., Barragan-Mendez C., Martinez-Rodriguez G., Mancera J.M. (2018). Gene expression of thyrotropin- and corticotrophin-releasing hormones is regulated by environmental salinity in the euryhaline teleost *Sparus aurata*. Fish Physiol. Biochem..

[21] Ranjbar M., Nejad M.M. (2020). Effect of water salinity on enzymatic and hormonal indices of rainbow trout (*Oncorhynchus mykiss*) fingerlings. Int. Aquat. Res..

[22] Bojarski B., Witeska M., Kondera E., Bartosz B. (2025). Blood biochemical biomarkers in fish toxicology—A review. Animals.

[23] Mozanzadeh M.T., Yaghoubi M., Marammazi J.G., Safari O., Gisbert E. (2018). Hemato-immunological and plasma biochemical responses of silvery-black porgy (*Sparidentex hasta*) fed protein and essential amino acid deficient diets. Comp. Clin. Pathol..

[24] Chen H.Q., Bi B.L., Hu Q. (2022). Effects of Salinity Acclimation on Flesh Quality, Serum Biochemical Indices and Na+-K+-ATPase Activity in Tilapia (*Oreochromis* spp.). J. Yunnan Agric. Univ. (Nat. Sci.).

[25] Rizzatti G., Lopetuso L.R., Gibiino G., Binda C., Gasbarrini A. (2017). Proteobacteria: A common factor in human diseases. BioMed Res. Int..

[26] Song L.Y., Cheng Y.M., Zhao J.L. (2020). Effects of salinity stress on hepatic fatty acid composition and lipid metabolism-related gene expression in Nile tilapia (*Oreochromis niloticus*). Chin. J. Fish..

[27] Tamrakar S., Kimmel J.G., Chung-Davidson Y.-W., Buchinger T.J., Scribner K.T. (2023). Determination of thyroid hormones in lake sturgeon (*Acipenser fulvescens*) tissues at different developmental stages using a sensitive liquid chromatography-mass spectrometry method. J. Chromatogr. B.

[28] Chang R.J.A., Celino-Brady F.T., Breves J.P., Seale A.P. (2025). Environmental salinity differentially impacts branchial and hepatic carbohydrate metabolism in tilapia. J. Fish Biol..

[29] Sadoul B., Geffroy B. (2019). Measuring cortisol, the major stress hormone in fishes. J. Fish Biol..

[30] van Muilekom D.R., Mueller J., Lindemeyer J., Schultheiß T., Maser E., Seibel H., Rebl A., Schulz C., Goldammer T. (2024). Salinity change evokes stress and immune responses in Atlantic salmon with microalgae showing limited potential for dietary mitigation. Front. Physiol..

[31] Dominguez M., Takemura A., Tsuchiya M. (2005). Effects of environmental factors on non-specific immune responses in Nile tilapia (*Oreochromis niloticus L.*). Aquac. Res..

[32] Sinha A.K., Liew H.J., Diricx M., Kumar V., Darras V.M. (2012). Combined effects of high environmental ammonia, starvation and exercise on hormonal and ion-regulatory response in goldfish (*Carassius auratus* L.). Aquat. Toxicol..

[33] Ferraris R.P., Almendras J.M., Jazul A.P. (1988). Changes in plasma osmolality and chloride concentration during abrupt transfer of milkfish (*Chanos chanos*) from seawater to different test salinities. Aquaculture.

[34] Zhao F., Wu B., Yang G., Zhang T., Zhuang P. (2016). Adaptive alterations on gill Nat, K+-ATPase activity and mitochondrion-rich cells of juvenile *Acipenser sinensis* acclimated to brackish water. Fish Physiol. Biochem..

[35] Chen J., Cai B., Tian C., Jiang D., Shi H., Huang Y., Zhu C., Li G., Deng S. (2023). RNA sequencing (RNA-Seq) analysis reveals liver lipid metabolism divergent adaptive response to low- and high-salinity stress in spotted scat (*Scatophagus argus*). Animals.

[36] Li Y.-Y., Hu C.-B., Zheng Y.-J., Xia X.-A., Xu W.-J. (2008). The effects of dietary fatty acids on liver fatty acid composition and Δ6-desaturase expression differ with ambient salinities in *Siganus canaliculatus*. Comp. Biochem. Physiol. Part B Biochem. Mol. Biol..

[37] Zhou T., Meng Q., Sun R., Xu D., Zhu F., Jia C., Zhou S., Chen S., Yang Y. (2024). Structure and gene expression changes of the gill and liver in juvenile black porgy (*Acanthopagrus schlegelii*) under different salinities. Comp. Biochem. Physiol. Part D Genom. Proteom..

[38] Martínez-Álvarez R.M., Sanz A., García-Gallego M., Domezain A., Domezain J., Carmona R., Ostos-Garrido M.D.V., Morales A.E. (2005). Adaptive branchial mechanisms in the sturgeon *Acipenser naccarii* during acclimation to saltwater. Comp. Biochem. Physiol. A.

[39] Maryoung L.A., Lavado R., Bammler T.K., Gallagher E.P., Stapleton P.L., Beyer R.P., Farin F.M., Hardiman G., Schlenk D. (2015). Differential gene expression in liver, gill, and olfactory rosettes of coho salmon (*Oncorhynchus kisutch*) after acclimation to salinity. Mar. Biotechnol..

[40] Rabeh I., Telahigue K., Gazali N., Chétoui I., Boussoufa D. (2013). Time course of changes in fatty acid composition in the osmoregulatory organs of the thicklip grey mullet (*Chelon labrosus*) during acclimation to low salinity. Mar. Freshw. Behav. Physiol..

[41] Jin R., Xia H., Yang P., Lu J., Chen F., Zhang Y., Liu L., Chen Z., Zeng H., Zhou W. (2023). Research progress on the fish complement C3 gene. Isr. J. Aquac.—Bamidgeh.

[42] Schmitz M., Mandiki S.N.M., Baekelandt S., L’Hoir M., Kestemont P. (2016). Chronic hyperosmotic stress interferes with immune homeostasis in striped catfish (*Pangasianodon hypophthalmus*, S.) and leads to excessive inflammatory response during bacterial infection. Fish Shellfish Immunol..

[43] Xu C., Li E., Suo Y., Su Y., Lu M. (2018). Histological and transcriptomic responses of two immune organs, the spleen and head kidney, in Nile tilapia (*Oreochromis niloticus*) to long-term hypersaline stress. Fish Shellfish Immunol..

[44] Wang S., Li M., Jiang Y., Sun C., Wu G. (2023). Transcriptome analysis reveals immune regulation in the spleen of koi carp during Aeromonas hydrophila infection. Mol. Immunol..

[45] Ahmed H., Bakry K.A., Abdeen A., El Bahgy H.E.K., Abdo M. (2023). The involvement of antioxidant, stress, and immune-related genes in the responsive mechanisms of common carp (*Cyprinus carpio*) to hypersalinity exposure. Front. Mar. Sci..

[46] Marcos-López M., Ruiz C.E., Rodger H.D., O’Connor I., MacCarthy E., Esteban M.Á. (2017). Local and systemic humoral immune response in farmed Atlantic salmon (*Salmo salar* L.) under a natural amoebic gill disease outbreak. Fish Shellfish Immunol..

[47] Shang X., Xu W., Zhang Y., Sun Q., Li Z. (2023). Transcriptome analysis revealed the mechanism of *Luciobarbus capito* (*L. capito*) adapting high salinity: Antioxidant capacity, heat shock proteins, immunity. Mar. Pollut. Bull..

[48] Brestoff J.R., Artis D. (2013). Commensal bacteria at the interface of host metabolism and the immune system. Nat. Immunol..

[49] Mkulo E.M., Iddrisu L., Yohana M.A., Zheng A., Zhong J., Jin M., Danso F., Wang L., Zhang H., Tang B. (2025). Exploring salinity adaptation in teleost fish, focusing on omics perspectives on osmoregulation and gut microbiota. Front. Mar. Sci..

[50] Gong J., Xu F., Li Y., He Y., Liang Z. (2024). Metagenomic analysis of intestinal microbial function and key genes responsive to acute high-salinity stress in Nile tilapia (*Oreochromis niloticus*). Gene.

[51] Lai K.P., Lin X., Tam N., Ho J.C.H., Wong M.K.-S. (2020). Osmotic stress induces gut microbiota community shift in fish. Environ. Microbiol..

[52] Jin G., Zhang L., Ai Q., Mai K., Chen X. (2025). Interactive effects of salinity and dietary lipid sources on growth, hepatic lipid metabolism, and transcriptomic profiles in spotted sea bass (*Lateolabrax maculatus*). Front. Physiol..

[53] Gillingham M.A.F., Prueter H., Montero B.K., Kempenaers B. (2025). The costs and benefits of a dynamic host microbiome. Trends Ecol. Evol..

[54] Liang X., Zhang Y., Ye T., Liu F., Lou B. (2025). Thyroid-Active Agents Triiodothyronine, Thyroxine and Propylthiouracil Differentially Affect Growth, Intestinal Short Chain Fatty Acids and Microbiota in Little Yellow Croaker *Larimichthys polyactis*. Fishes.

[55] Wu S.G., Pan M.J., Zan Z.Y., Jakovlić I., Zhao W., Zou H., Ringø E., Wang G. (2023). Regulation of lipid metabolism by gut microbiota in aquatic animals. Rev. Aquac..

[56] Cui J.R., Lai Q.F., Li Y.-H. (2026). Mechanisms of fish intestinal damage under environmental stress. Insights From Carbonate Saline–Alkali Water Perspective. Rev. Aquac..

